# Deciphering the Potential of Pre and Pro-Vitamin D of Mushrooms against Mpro and PLpro Proteases of COVID-19: An In Silico Approach

**DOI:** 10.3390/molecules27175620

**Published:** 2022-08-31

**Authors:** Abhay Tiwari, Garima Singh, Gourav Choudhir, Mohit Motiwale, Nidhi Joshi, Vasudha Sharma, Rupesh K. Srivastava, Satyawati Sharma, Marco Tutone, Pradeep Kumar Singour

**Affiliations:** 1 Centre for Rural Development & Technology, Indian Institute of Technology (IIT), New Delhi 110016, India; 2 Computational and Synthetic Chemistry Lab., Department of Pharmaceutical Chemistry, Faculty of Pharmacy, VNS Group of Institutions, Bhopal 462044, India; 3 Department of Pharmacology, University of Minnesota, Minneapolis, MN 55455, USA; 4 Department of Food Technology, Jamia Hamdard, New Delhi 110062, India; 5 Department of Biotechnology, All India Institute of Medical Sciences (AIIMS), New Delhi 110029, India; 6 Dipartimento di Scienze e Tecnologie Biologiche Chimiche e Farmaceutiche, Università degli Studi di Palermo, 90123 Palermo, Italy

**Keywords:** edible mushrooms, SARS-CoV-2, pre-vitamin-D, pro-vitamin-D, in-silico studies

## Abstract

Vitamin D’s role in combating the SARS-CoV-2 (severe acute respiratory syndrome coronavirus 2), the virus causing COVID-19, has been established in unveiling viable inhibitors of COVID-19. The current study investigated the role of pre and pro-vitamin D bioactives from edible mushrooms against Mpro and PLpro proteases of SARS-CoV-2 by computational experiments. The bioactives of mushrooms, specifically ergosterol (provitamin D_2_), 7-dehydrocholesterol (provitamin-D_3_), 22,23-dihydroergocalciferol (provitamin-D_4_), cholecalciferol (vitamin-D_3_), and ergocalciferol (vitamin D_2_) were screened against Mpro and PLpro. Molecular docking analyses of the generated bioactive protease complexes unravelled the differential docking energies, which ranged from −7.5 kcal/mol to −4.5 kcal/mol. Ergosterol exhibited the lowest binding energy (−7.5 kcal/mol) against Mpro and PLpro (−5.9 kcal/mol). The Molecular Mechanics Poisson–Boltzmann Surface Area (MMPBSA) and MD simulation analyses indicated that the generated complexes were stable, thus affirming the putative binding of the bioactives to viral proteases. Considering the pivotal role of vitamin D bioactives, their direct interactions against SARS-CoV-2 proteases highlight the promising role of bioactives present in mushrooms as potent nutraceuticals against COVID-19.

## 1. Introduction

The pandemic predicament of COVID-19 has devastated the entire world. The unprecedented contagion of coronavirus (CVs) has entirely changed the world’s economic, political, and social status [1]. COVID-19 is initially caused by severe acute respiratory syndrome coronavirus 2 (SARS-CoV-2) [2]. SARS-CoV-2, with an approximate size of 26–32 kb contains an RNA (single-stranded) genome encased in a glycoside spike-covered enclosing membrane. Phylogenetic studies have shown that the SARS-CoV is a member of the Beta coronavirus genus [3].

As per the data reported by the Johns Hopkins Coronavirus Resource Center, more than 6 million deaths due to CVs have been reported up to 20 May 2022 (https://coronavirus.jhu.edu/map.html; accessed on 20 May 2022). This epidemic situation continues, and due to the incessant mutation of SARS-CoV-2, different variants of CoV-2 (α, β, γ, δ, omicron, etc.) have been reported across the globe. These statistics raise stringent apprehension among people about the lack of effective medicinal recourse and treatments against CVs [4,5]. However, effective measures to reduce the magnitude of the disease are confined to supportive techniques to prevent the consequences of CVs. Several drug-discovery methodologies, such as quantitative structure–activity relationship (QSAR), artificial intelligence, drug repositioning, and virtual screening (VS), are critical for unveiling a therapy for the uncontrollable COVID-19 pandemic [6,7,8].

Recently, the outbreak of COVID-19 shattered the entire world and dramatically impacted the social and economic situation globally. Despite the overall positiveness, the COVID-19 pandemic still dominates as the most notable risk to economic growth in respondents’ countries. The pandemic is predominantly caused by severe acute respiratory syndrome coronavirus 2 (SARS-CoV-2). The virus causes severe hyperinflammation of the lungs and leads to acute respiratory distress, ultimately leading to death. Although several researchers have suggested a combination of antiviral and anti-inflammatory drug-based therapies, the effective treatment of COVID-19 is still arguable. Various natural compounds with more efficiency and less toxicity have been utilised to treat the disease; compounds obtained from mushrooms have promisingly gained attention as alternative medicine. They have astonishing anti-HIV protease and anti-inflammatory activities that have established these compounds as an alternative strategy for treating COVID-19 [9].

Exploring and illuminating the SARS-CoV-2 targets has become a crucial step in molecular investigations. In this regard, the main SARS-CoV-2 protease (Mpro) and its papain-like protease (PLpro), essential elements of the virus’s infectious pathway, have gained alluring attention as effective targets for therapeutic efforts. Numerous studies have proclaimed that people with vitamin D deficiency have a higher potential to develop critical cases of COVID-19 than individuals who maintain adequate vitamin D levels in their blood [10]. The administration of oral doses of vitamin D and mineral supplementation could fulfil our bodies’ demands for vitamin D. However, cultural taboos (for example, vegans) and vegetarian diets could restrict vitamin D-rich dietary options.

Polyproteins 1a and 1ab (pp1a and pp1ab) are two overlapping translation products encoded by the SARS-CoV-2 replicase gene (Orf1), which regulate all the critical processes required for viral replication. The auto-cleavage of the polyproteins pp1a and pp1ab releases the major protease (Mpro), a crucial enzyme in viral replication. Mpro then cleaves pp1a and pp1ab, releasing the functional proteins nsp4 to nsp16, which are required for viral replication. Furthermore, the proteolytic cleavage of the viral polyprotein precursors pp1a and pp1ab at three locations by the cysteine protease papain-like protease (PLpro) forms the non-structural proteins Nsp1, Nsp2, and Nsp3. As a result, Mpro and PLpro are equally critical in virus transcription and replication and innovative antiviral medication design [11].

Concerning the requirement for vitamin D intake in COVID-19, the use of mushrooms has earned extensive awareness as a potential source of natural vitamin D and other valuable bioactive compounds [12,13]. Consuming mushrooms that are vitamin D-enriched is safe and provides vitamin D in all of its isoforms. Several organisations, including the FDA and EFSA, have already released rules pertaining to the creation and development of fortified foods based on irradiated vitamin D-enriched mushrooms [14,15]. The benefits of mushroom products on many clinical disorders have drawn substantial attention from the scientific community in the past decade. The perception of mushrooms as highly nutritional foodstuffs is well established, and consequently, they are considered beneficial food supplements with several valuable bioactive compounds exhibiting enormous therapeutic potential [15].

One of the prevailing conditions linked to high death rates in COVID-19 patients is vitamin D deficiency [10]. Notably, due to poor exposure of individuals to sunlight in certain seasons and at certain latitudes and the social restraints enforced as part of the pandemic plan, vitamin D deficiency may afflict many people, especially the aged. The blood’s low vitamin D levels have been coupled to various metabolic disorders. Clinical experience has indicated that these factors increase the risk of a severe COVID-19 illness course. Although multiple studies and investigations have revealed a link between vitamin D deficiency and COVID-19 illness and death intensity [16,17], no molecular/structural information is available concerning its direct involvement in the disease.

Mushrooms are the only source of pre- and pro-vitamin Ds in which other bioactives, such as polysaccharides, terpenes, β-glucans, ergothioneine, lectins, polyphenols, etc., are also present; this makes them a unique natural source of valuable bioactives with various activities, including immunostimulant, immunomodulating, antiviral, anticancer, and antidiabetic activity [12,17,18,19,20,21,22,23]. Studies by Rangsinth et al. (2021) and Elhusseiny et al. (2022) have indicated the efficacy of different mushroom bioactives against the main protein of SARS-CoV-2 [9,24]. Copious studies have also shown a putative link between vitamin D levels and COVID-19 infection [25,26,27,28,29]; this motivated us to unravel the potential of pre- and pro-vitamin Ds present in the different edible mushroom species against Mpro and PLpro proteases of COVID-19 through computational (molecule docking and molecular dynamics simulation) methods. As no in silico studies are available on this subject, the current study represents unique research and the principal in silico investigation.

## 2. Materials and Methods

### 2.1. Molecular Docking and Ligand Preparation

The 3D structure of pre- and pro-vitamin Ds present in mushrooms was retrieved using the PubChem database (https://pubchem.ncbi.nlm.nih.gov; accessed on 23 May 2022). The generated structures were saved in PDB format with Open Babel software [30]. All the molecules were permitted to go through energy reduction and optimisation utilising the universal force field (UFF) at the 200 descent steepest method of PyRx’s Open Babel tools. The generated files were saved in pdbqt format. The Mpro structure (PDB ID: 5R82) and the PLpro structure (PDB ID: 7JRN) were used in the present study. UCSF Chimera software was used to exclude water and other non-specific compounds [31]. Polar hydrogens were added to protein protonation to preserve cellular pH (pH = 7). The side chain angles were corrected using Discovery Studio’s clean geometry tool (San Diego, CA, USA).

Molecular docking of pre- and pro-vitamin Ds was performed as per the methodology of Trott and Olson, 2010, using the software AutoDock Vina [32]. The grid centre point was assigned the coordinates x (9.194), y (−0.252), and z (21.440) for Mpro. For PLpro, the coordinates allocated were x (19.811), y (−4.828), and z (−25.36). The dimensions of the box were assigned as 24 Å × 24 Å × 24 Å with an exhaustiveness of 8 for both cases. Using AutoDock MGL Tools v1.5.6, the Kollman charges (−30.252 for Mpro and −15.28 for PLpro) and hydrogen atoms were added to the protein structure (The Scripps Research Institute, Molecular Graphics Laboratory, 10550 North Torrey Pines Road, 92037, San Diego, CA, USA). The protein obtained was stored as a pdbqt file. Open Babel was used to create the pdbqt file format for all ligands. The default settings for other docking parameters were used. The images of the molecular docking interactions were created using the Biovia Discovery Studio Visualizer 2019. The docking protocol can be validated by performing a redocking experiment. Before and after docking, the root-mean-square deviation (RMSD) of the co-crystal structures should be less than 2.0 Å [33,34,35].

### 2.2. Molecular Dynamics (MD) Simulations

MD simulations were performed for the generated complexes to determine their flexibility and stability. The GROMOS96 43a1 force field was applied to all the simulations performed using the GROMACS 5.1.4 package [36]. The ligand topology files were produced using the PRODRG server [37]. SPC water molecules were employed to solvate the produced protein complexes using a 10 nm edge length in a cubic box. To keep the solution neutral, the correct number of ions was introduced. Energy minimisation calculations were performed with a convergence threshold of 1000 kJ/mol/nm to reduce conflicts between the atoms in the system. The Particle Mesh Ewald (PME) evaluated the electrostatic interactions of long-range macromolecules [38].

A 9 Å cut-off radius was employed for the Van der Waals and coulombic contacts. The simulation was performed with two phases of equilibration. The solvent and ions were left unrestrained in the first phase of the NPT ensemble, while the constraint weight of protein and protein–ligand complexes was gradually dropped in the second phase. The LINCS algorithm was employed to restrict the hydrogen bonds [39,40]. The system’s pressure and the temperature were kept at 1 atm and 300 K, respectively, achieved by coupling the Parrinello–Rahman pressure and Berendsen’s temperature [41]. The simulation was launched by executing the velocity and coordinates acquired following the final phase of the equilibration procedure. For all systems, the simulation time was 100 ns, and snapshots were collected every two picoseconds (ps).

### 2.3. MM/PBSA Free Energy Calculation

The protein–ligand complexes’ binding energies were calculated using the MM-PBSA (Molecular Mechanics Poisson–Boltzmann Surface Area) by implementing the following equations:

(1) $$\Delta G=\langle \Delta G_{PL}\rangle -\left(\langle G_{p}\rangle +\langle G_{L}\rangle \right)$$

ΔG: binding energy; $\langle \Delta G_{PL}\rangle$: free energies of the complex expressed as the mean; $\langle G_{p}\rangle$ and $\langle G_{L}\rangle$: receptor and ligand-free energies, respectively.

Furthermore, Equation (1) can be expressed as:

(2) $$\Delta G=\Delta E_{MM}+\Delta G_{psolv}+\Delta G_{npsolv}-T\Delta S$$

$\Delta E_{MM}$: molecular mechanics interaction energy; $\Delta G_{psolv}$ and $\Delta G_{npsolv}$: solvation energy of polar and nonpolar molecules, respectively; T: temperature; S: entropy.

$\Delta G_{psolv}$ was computed through the Poisson–Boltzmann equation, and $\Delta G_{npsolv}$ was estimated by exploiting the linear relationship between the polar part of the solvent-accessible surface area and the solvation energy.

The salt ionic strength and solute dielectric constants were set at 4.0 M and 0.15 M, respectively. The binding free energy was assessed by employing the single-trajectory procedure. The g_mmpbsa module of GROMACS was employed to calculate the binding free energy of complexes as per the methodology of Kumari et al. [42]. We discovered stable compounds and used the final ten nanoseconds of data for analysis, and snapshots were taken at every 100 picoseconds. The entropy term −TΔS was not calculated to reduce computational time, as previously reported [43,44,45].

## 3. Results and Discussion

### 3.1. Molecular Docking Analysis

Pre- and pro-vitamin Ds molecules (the 2D structures of the compounds are given in Supplementary Figure S1) were docked against the potential target proteins of COVID-19. The docking results for SARS-2 Mpro revealed that pre- and pro-vitamin D molecules’ binding energies were in the range of −7.5 to −6.3 kcal/mol (Table 1).

The docking results for SARS-2 PLpro indicated that the molecules’ binding energies were distributed in the −5.9 to −4.5 kcal/mol range (Table 1). Binding energies depend on non-covalent intermolecular interactions and the number of residues present within the protein and ligand binding region. The ligand binds with Mpro covering many residues (>10) that are also involved in interactions. At the same time, PLpro has a smaller number of residues (<10) present within the ligand’s binding region and forms fewer interactions. This signifies the plausible reason for the difference in the bonding energies obtained for PLpro and Mpro.

The binding energies of all the pre- and pro-vitamin D molecules established their pronounced involvement to serve as appropriate bioactives against SARS-CoV-2 Mpro and PLpro.

**Figure 1:**
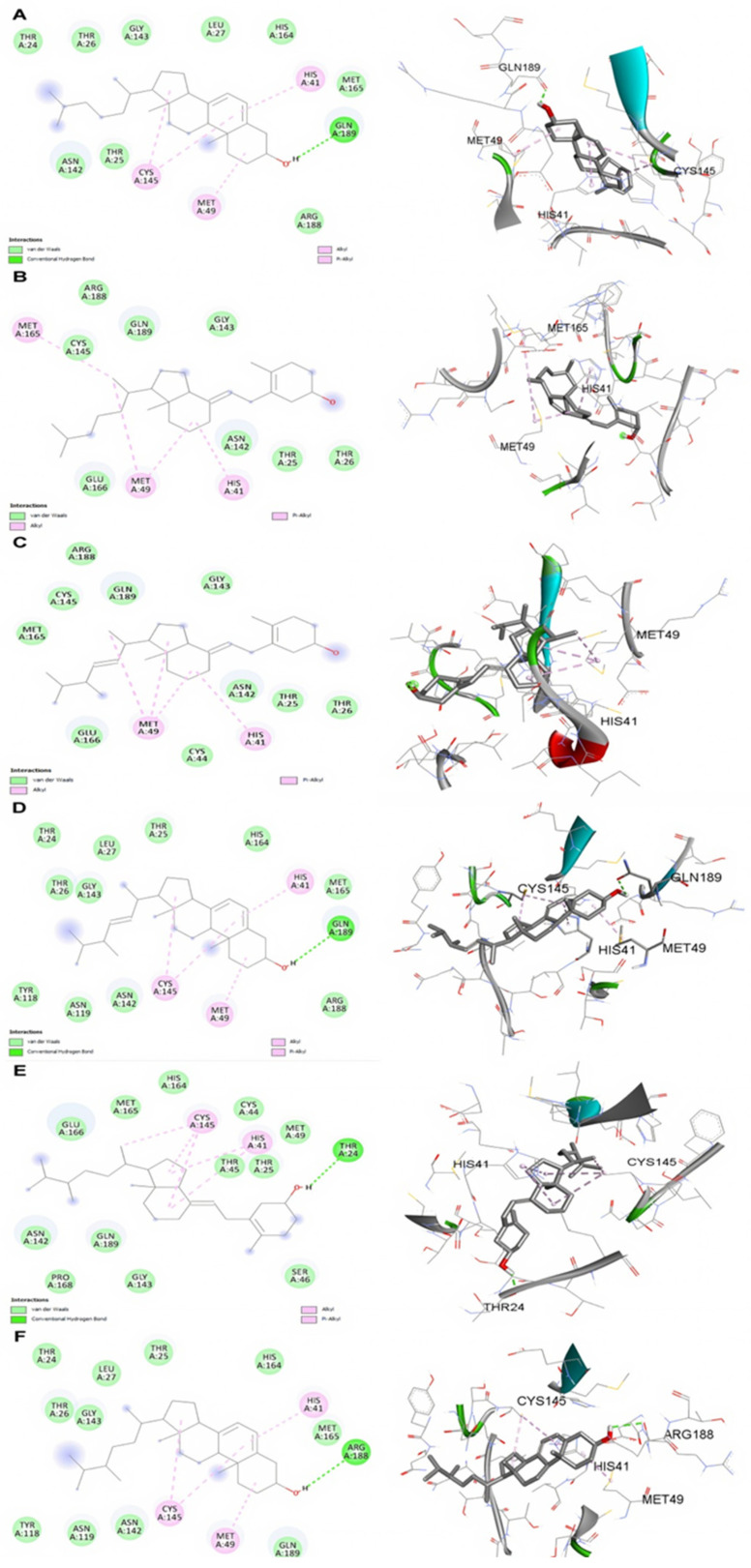
Molecular docking 2D and 3D interaction representation between SARS-2 Mpro and (**A**) 7-dehydrocholesterol, (**B**) cholecalciferol, (**C**) ergocalciferol, (**D**) ergosterol, (**E**) 22,23-dihydroergocalciferol, and (**F**) 22,23-dihydroergosterol.

**Figure 2:**
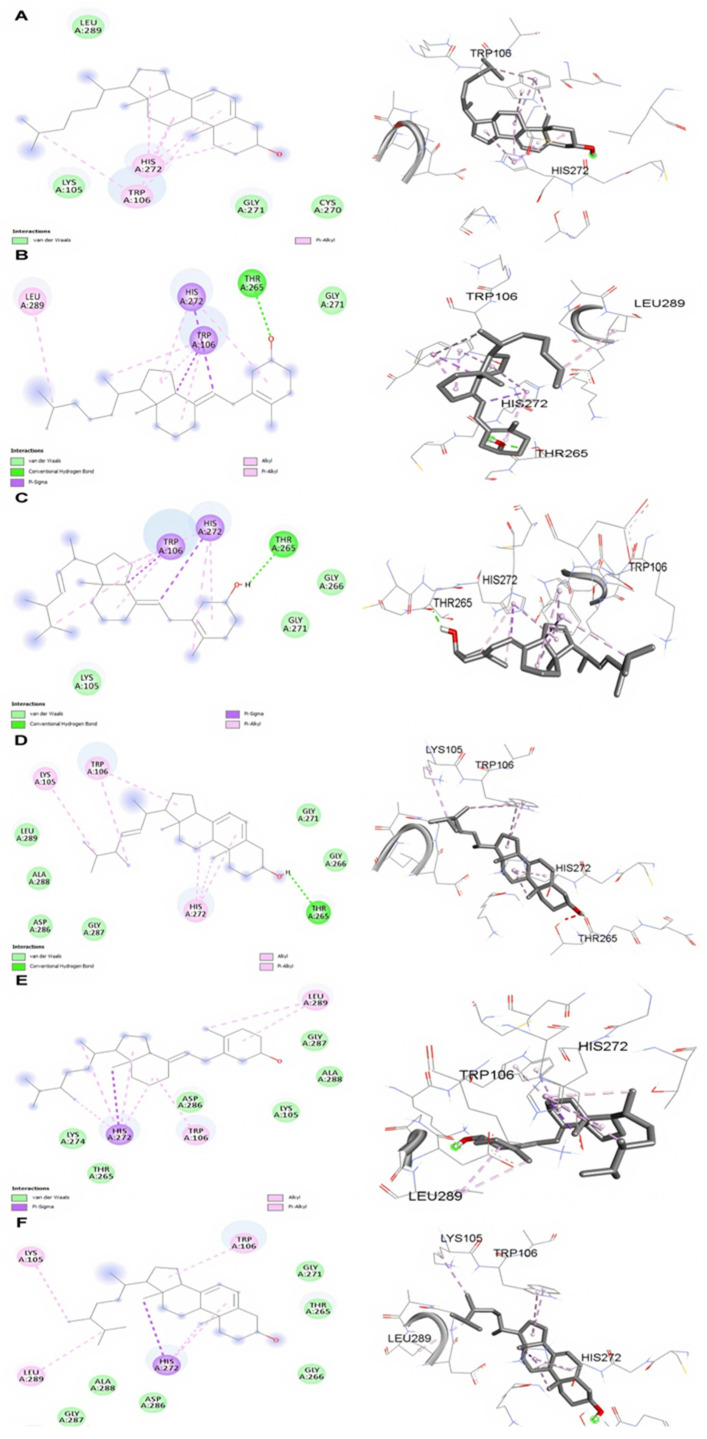
Molecular docking 2D and 3D interaction representation between SARS-2 PLpro and (**A**) 7-dehydrocholesterol, (**B**) cholecalciferol, (**C**) ergocalciferol, (**D**) ergosterol, (**E**) 22,23-dihydroergocalciferol, and (**F**) 22,23-dihydroergosterol.

The two-dimensional-interaction of the docked molecules, as shown in Figure 1 and Figure 2, depicted that in the case of SARS-CoV-2 Mpro, the screened pre- and pro-vitamin Dmolecules cholecalciferol and ergocalciferol formed no hydrogen bond interactions (Figure 1B,C), while 22,23-dihydroergocalciferol formed a hydrogen bond with THR24, and 22,23-dihydroergosterol formed a hydrogen bond with ARG189 (Figure 1E,F). Interestingly, 7-dehydrocholesterol and ergosterol formed hydrogen bonds with GLN189 (Figure 1A,D).

The docking results of SARS-CoV-2 PLpro unveiled that the three molecules, i.e., ergosterol, cholecalciferol, and ergocalciferol, formed hydrogen bonds with the THR265 amino acid residue, while no hydrogen bonding was observed with the other molecules of pre- and pro-vitamin D molecules (Figure 2A–F). The molecular docking study was validated with the help of the redocking experiment. The original and docked conformations of co-crystallised ligands of Mpro and PLpro are shown in Figure. The RMSD between the two conformations were found to be 0.512 Å and 0.416 Å for Mpro and PLpro, respectively (Supplementary Figure S2).

### 3.2. Protein–Ligand Complex Conformational Dynamics and Stability

**Figure 3:**
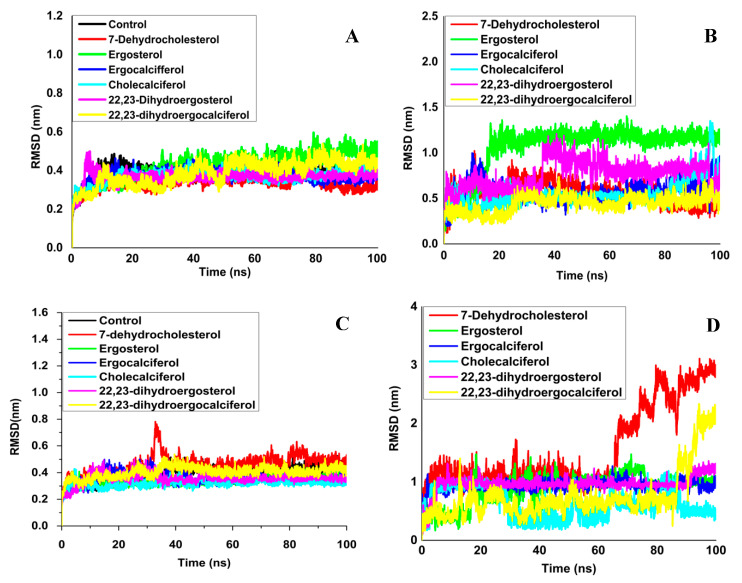
The root-mean-square deviation (RMSD) analysis. (**A**) The RMS deviation of Mpro in the apo and ligand-bound states. (**B**) The RMS deviation of ligands coupled to Mpro’s active site. (**C**) The RMS deviation of PLpro in the apo and ligand-bound states. (**D**) The RMS deviation of ligands coupled to the active site of PLpro.

To comprehend the conformational stability and temporal evolution changes of Mpro and Mpro–ligand complexes, as well as those of PLpro and PLpro–ligand complexes, RMSD was performed. The RMSD trajectory of Mpro and PLpro swiftly reached a stable equilibrium within the first ten nanoseconds of simulation, as shown in Figure 3. The protein structure was stable, with an average variation of 0.388 ± 0.042 nm in Mpro and 0.391 ± 0.063 nm in PLpro (Table 2). The RMSD profile for the Mpro–ligand MD simulation ranged from 0.444 to 1.062 nm, while for PLpro–ligand, it ranged from 0.326 to 0.483 nm (Table 2). Figure 3A (Mpro) and Figure 3C (PLpro) show a similar trend in terms of making stable complexes. The RMS distribution of all ligands’ apo and holo forms was acute and uni-modal, with 0.3–0.4 nm peaks. The RMSDs of ligands for Mpro (Figure 3B) and PLpro (Figure 3D) were further evaluated with the inactive ligand–protease site to capture the conformational dynamics.

Unlike Mpro and PLpro, the inhibitors possessed a substantial RMS deviation (Table 2). For Mpro, the highest RMSD of 1.062 nm was demonstrated by ergosterol, whereas 7-dehydrocholesterol showed the highest RMSD of 1.537 nm for PLpro, indicating the flexibility of ligand binding in the active sites of Mpro and PLpro. Kumar et al. (2021) [46] and Motiwale et al. (2020) [47] documented a similar pattern of RMSD analysis for phytochemical inhibitors against SARS Mpro.

**Figure 4:**
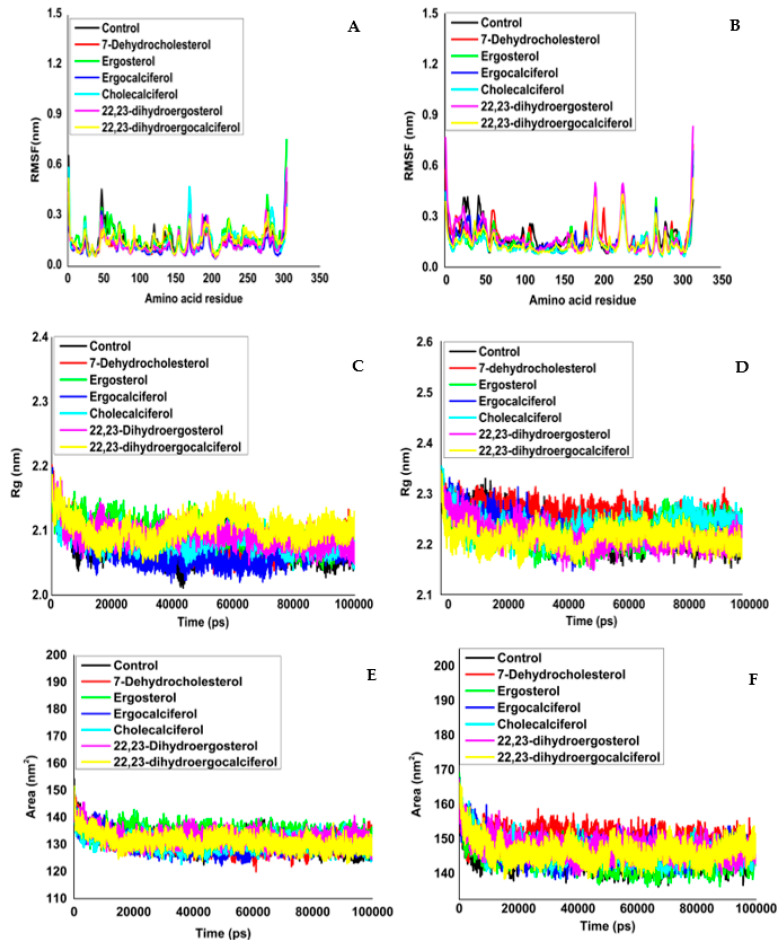
Root-mean-square fluctuations (RMSF) from the initial structures of (**A**) the main protease and main protease–ligand complexes and (**B**) papain-like protease and papain-like protease–ligand complexes during the simulation time. Time evolution plot of Rg for all Cα atoms in apo and holo states of (**C**) Mpro and (**D**) PLpro. Solvent accessible surface area (SASA) of (**E**) Mpro and complex and (**F**) PLpro and complex.

RMSF (root-mean-square fluctuation) analysis was executed to demonstrate the local and conformational dynamics of Mpro and PLpro in free and ligand-bound form. The individual amino acid’s average fluctuation was calculated. Figure 4A and Table 3 (Mpro) and Figure 4B and Table 3 (PLpro) illustrated the RMSF variation of all Cα atoms. Most residual fluctuations had an average strength of 0.1 to 0.4 nm. However, only a few amino acids in the C and N terminus were linked to significant RMSF scores (>0.4 nm in Mpro and >0.6 nm in PLpro). By contrast, the amino acids in the α-helix and β-sheet were characteristically stable. The imbricating trend in RMSF implied that ligand binding imposed no considerable impact on amino acid location.

The time-dependent fluctuation of Rg (radius of gyration) was estimated to establish the influence of ligand binding on the compactness and structural integrity of Mpro and PLpro. Figure 4 reflects that the average difference in Rg in the free form in Mpro (Figure 4C, Table 4) was 2.077 ± 0.020 nm, while in PLpro, it was 2.225 ± 0.031 nm (Figure 4D, Table 4). The stable radius of the protease gyration chart suggested that Mpro’s structural integrity was preserved throughout the simulation. Subsequently, in the protease–ligand complexes, Rg variation oscillated from 2.066–2.104 nm and 2.213–2.257 nm range for Mpro and PLpro, respectively.

Solvent accessible surface area (SASA) assessment determines the protein’s accessibility to a solvent. The SASA results for specific amino acids in the holo and apo form of protein are graphically depicted in Figure 4e (Mpro) and f (PLpro). Residues of Mpro and PLpro in free and ligand-bound form showed sharp peaks from 130.364 to132.702 nm^2^ and 144.742 to 149.943 nm^2^, respectively, and they are outlined in Table 5 and Figure 4. The results corroborate that the ligand interaction does not lead to any structural alterations in Mpro and PLpro’s active sites.

### 3.3. Assessment of Binding Free Energy and Hydrogen Bond Analysis

In the conformational dynamics studies, it was observed that the ligand-binding events did not impact the primary protease’s structural integrity. Binding free energy was calculated for Mpro and PLpro using MM/PBSA analysis to assess the impact on the proteases’ active sites upon binding the respective inhibitors. Table 6 and Table 7 list the binding free energies for all the bioactives, which varied from −136.116 ± 14.005 to −186.019 ± 14.239 in Mpro and −87.413 ± 18.585 to −197.157 ± 14.673 in PLpro.

**Figure 5:**
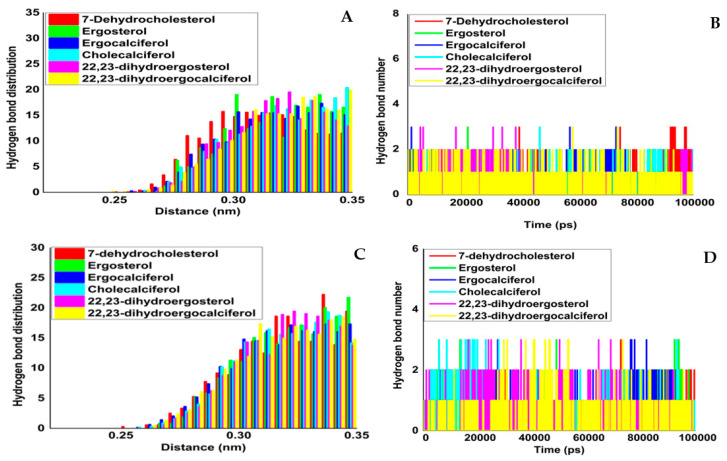
Hydrogen bond formation during the 100 ns simulation and hydrogen bond distribution between protease and ligand between Mpro and ligand molecules. (**A**) Hydrogen bond distribution; (**B**) no. of hydrogen bonds, PLpro and ligand molecules; (**C**) hydrogen bond distribution, and (**D**) no. of hydrogen bonds.

Hence, the results suggest that the bioactive cholecalciferol contained the lowest binding energy of −136.116 ± 14.005 against Mpro. By contrast, ergocalciferol had the lowest binding free energy of −96.248 ± 49.375 against PLpro, indicating positive interactions. Notably, the binding free energy was profoundly influenced by the polar solvation (ΔGpsolv) and Van der Waals (ΔEvdw) interactions. Furthermore, hydrogen bonding maps were also analysed to comprehend the active sites’ spatial interactions between inhibitors and proteases (major and papain-like) (Figure 5A–D and Table 8).

Hydrogen bonds are essential in determining the interaction strength among enzymes and ligands. The temporal dependence of intermolecular hydrogen bonding between receptor and drug-like entities was investigated during the simulation period to predict the binding of pre- and pro-vitamin D molecules in the active site (MPro and PLPro). During the 100 ns simulation run, the ligand–protein made multiple hydrogen bonds, according to the hydrogen bond analysis. The hydrogen bonds were scattered at 0.25–0.35 nm, according to the hydrogen bond pattern depicted in Figure 5.

## 4. Conclusions

The Mpro and PLpro proteases of COVID-19 have been identified as decisive and effective targets in inhibiting coronavirus replication. Hence, the present work tried to unveil the potential role of pre-and pro-vitamin Ds of edible mushrooms against COVID-19 through in silico studies. These compounds are considered to potentially inhibit the main SARS-CoV-2 protease and may become helpful in treating COVID-19 patients. The pronounced observations of the current research conferred that edible mushrooms have enormous potential in squelching Mpro and PLpro proteases and are in good agreement with the suggested role of these compounds in inhibiting the COVID-19 virus. Thus, due to mushroom compounds’ various prominent activities against coronavirus, the current study strongly supports using them for COVID-19 treatment.

Furthermore, the study also suggests that incorporating this vitamin D-rich mushroom-fortified food product into regular diets would help strengthen the body against viral infection. Using such compounds with very low or no toxicity would allow us to develop them as anti-COVID-19 drugs. In conclusion, even though this theoretical study needs to be confirmed by wet lab experiments to be planned, it opens the door to seeking novel insights for effectively using vitamin D-enriched mushrooms to combat COVID-19.

## Figures and Tables

**Table 1:** Details of ligand molecules along with binding energies.

Metabolites	PubChem ID	Molecular Formula	Molecular Weight	Binding Energy (kcal/mol)	
				M^Pro^	PL^Pro^
Ergocalciferol (pre-vitamin D_2_)	CID_5280793	C_28_H_44_O	396.6	−6.7	−5.9
Ergosterol (pro-vitamin D_2_)	CID_444679	C_28_H_44_O	396.6	−7.5	−5.1
Cholecalciferol (pre-vitamin D_3_)	CID_5280795	C_27_H_44_O	384.6	−6.3	−5.3
7-Dehydrocholesterol (pro-vitamin D_3_)	CID_439423	C_27_H_44_O	384.6	−6.5	−5.5
22,23-Dihydroergocalciferol (pre-vitamin D_4_)	CID_5460703	C_28_H_46_O	398.7	−6.6	−4.5
22,23-Dihydroergosterol (pro-vitamin D_4_)	CID_5326970	C_28_H_46_O	398.7	−7.1	−5

**Table 2:** Mean and standard deviation of RMSD of Mpro and PLpro in apo and ligand-bound states.

Name of Compound	M^pro^			PL^pro^		
	Backbone RMSD (nm)	Complex RMSD (nm)	Ligand RMSD (nm)	Backbone RMSD (nm)	Complex RMSD (nm)	Ligand RMSD (nm)
Control (Protein)	0.38 ± 0.04			0.39 ± 0.06		
7-Dehydrocholesterol	0.33 ± 0.03	0.34 ± 0.03	0.56 ± 0.12	0.45 ± 0.07	0.48 ± 0.08	1.53 ± 0.69
22,23-Dihydroergocalciferol	0.39 ± 0.06	0.39 ± 0.06	0.44 ± 0.08	0.40 ± 0.04	0.41 ± 0.04	0.75 ± 0.41
22,23-Dihydroergosterol	0.36 ± 0.03	0.37 ± 0.03	0.76 ± 0.14	0.35 ± 0.03	0.36 ± 0.04	0.96 ± 0.14
Cholecalciferol	0.36 ± 0.03	0.37 ± 0.03	0.54 ± 0.14	0.32 ± 0.02	0.32 ± 0.02	0.62 ± 0.24
Ergocalciferol	0.37 ± 0.03	0.37 ± 0.03	0.56 ± 0.11	0.35 ± 0.04	0.36 ± 0.04	0.93 ± 0.09
Ergosterol	0.46 ± 0.07	0.43 ± 0.07	1.06 ± 0.25	0.33 ± 0.03	0.34 ± 0.03	0.88 ± 0.25

**Table 3:** Root mean square fluctuations (RMSF) from the initial structures of the main protease and main protease-ligand complexes and papain-like protease and papain-like protease-ligand complexes.

Name	RMSF (nm)	
	M^Pro^	PL^Pro^
Control (protein)	0.16 ± 0.07	0.17 ± 0.08
7-Dehydrocholesterol	0.13 ± 0.06	0.17 ± 0.08
22,23-dihydroergocalciferol	0.15 ± 0.05	0.14 ± 0.06
22,23-dihydroergosterol	0.13 ± 0.06	0.17 ± 0.10
Cholecalciferol	0.14 ± 0.07	0.13 ± 0.07
Ergocalciferol	0.12 ± 0.06	0.16 ± 0.07
Ergosterol	0.17 ±0.07	0.15 ± 0.06

**Table 4:** Mean and standard deviation of Rg of Mpro and PLpro in apo and ligand-bound states.

Molecule ID	Rg (nm)	
	M^Pro^	PL^Pro^
Control (Protein)	2.07 ± 0.02	2.22 ± 0.03
7-Dehydrocholesterol	2.08 ± 0.02	2.25 ± 0.02
22,23-Dihydroergocalciferol	2.10 ± 0.01	2.21 ± 0.01
22,23-Dihydroergosterol	2.09 ± 0.01	2.21 ± 0.02
Cholecalciferol	2.08 ± 0.01	2.23 ± 0.02
Ergocalciferol	2.06 ± 0.02	2.23 ± 0.02
Ergosterol	2.09 ± 0.02	2.22 ± 0.02

**Table 5:** Solvent accessible surface area (SASA) of Mpro and complex.

Molecule ID	SASA (nm^2^)	
	M^Pro^	PL^Pro^
Control (Protein)	131.08 ± 3.23	144.26 ± 2.63
7-Dehydrocholesterol	130.77 ± 2.99	149.94 ± 2.54
22,23-Dihydroergocalciferol	131.21 ± 2.83	146.68 ± 3.27
22,23-Dihydroergosterol	132.70 ± 2.78	147.45 ± 2.78
Cholecalciferol	130.92 ± 2.50	146.56 ± 3.15
Ergocalciferol	130.36 ± 3.10	146.61 ± 3.26
Ergosterol	134.19 ± 2.44	144.74 ± 3.25

**Table 6:** MM/PBSA binding free energy (in kJ/mol) for vitamin D isoforms binding to SARS-CoV-2 main protease.

Molecule	ΔE_vdw_	ΔE_EEL_	ΔG_psolv_	ΔG_SASA_	ΔG_bind_
7-Dehydrocholesterol	−213.90 ± 10.71	−4.08 ± 2.55	63.71 ± 11.76	−18.56 ± 1.07	−172.83 ± 13.57
22,23-Dihydroergocalciferol	−166.70± 13.61	0.80 ± 1.64	37.57 ± 6.59	−15.86 ± 1.26	−144.19 ± 13.24
22,23-Dihydroergosterol	−213.34 ± 13.49	−3.16 ± 3.42	47.91 ± 7.45	−17.42 ± 1.10	−186.01 ± 14.23
Cholecalciferol	−164.82 ± 15.01	−0.23 ± 1.33	44.44 ± 10.42	−15.49 ± 1.49	−136.11 ± 14.00
Ergocalciferol	−177.43 ± 11.91	−0.15 ± 2.35	36.84 ± 7.68	−16.42 ± 1.15	−157.16 ± 14.03
Ergosterol	−252.04 ± 9.94	−5.74 ± 1.76	73.30 ± 10.94	−19.38 ± 0.89	−203.86 ± 13.02

**Table 7:** MM/PBSA binding free energy (in kJ/mol) for vitamin D isoforms binding to SARS-CoV-2 papain-like protease.

Molecular ID	ΔE_vdw_	Δ_EEEL_	ΔG_psolv_	ΔG_SASA_	ΔG_bind_
7-Dehydrocholesterol	−153.04 ± 6.91	−3.56 ± 4.23	38.97 ± 12.48	−13.67 ± 1.53	−131.31 ± 16.97
22,23-Dihydroergocalciferol	−106.94 ± 5.96	0.22 ± 2.10	30.46 ± 13.05	−11.15 ± 1.99	−87.41 ± 18.58
22,23-Dihydroergosterol	−143.53 ± 4.19	−15.90 ± 4.45	38.81 ± 21.38	−11.91 ± 1.73	−132.53 ± 22.19
Cholecalciferol	−123.46 ± 11.46	−0.41 ± 1.05	26.82 ± 5.33	−12.06 ± 1.11	−109.09 ± 11.53
Ergocalciferol	−122.07 ± 7.73	−0.006 ± 2.12	38.00 ± 38.05	−12.17 ± 2.83	−96.24 ± 49.37
Ergosterol	−239.58 ± 16.68	0.21 ± 3.22	62.29 ± 10.14	−20.08 ± 1.16	−197.15 ± 14.67

**Table 8:** Hydrogen bond distribution for Mpro and PLpro ligand molecules.

Molecule	M^pro^		PL^pro^	
	Donor–Acceptor Distance (nm)	Time (ns)	Dono—Acceptor Distance (nm)	Time (ns)
7-Dehydrocholesterol	2.857 ± 5.464	0.284 ± 0.51	2.857 ± 5.907	0.071 ± 0.276
22,23-Dihydroergocalciferol	2.857 ± 5.880	0.142 ± 0.384	2.857 ± 5.797	0.092 ± 0.400
22,23-Dihydroergosterol	2.857 ± 5.852	0.142 ± 0.393	2.857 ± 6.012	0.143 ± 0.400
Cholecalciferol	2.857 ± 5.856	0.130 ± 0.363	2.857 ± 5.911	0.139 ± 0.400
Ergocalciferol	2.857 ± 5.675	0.143 ± 0.383	2.857 ± 5.757	0.127 ± 0.359
Ergosterol	2.857 ± 5.876	0.048 ± 0.232	2.857 ± 6.046	0.095 ± 0.327

## Data Availability

All data generated or analysed during this study are included in this published article.

## Supplementary Materials

The following are available online at https://www.mdpi.com/article/10.3390/molecules27175620/s1, Supplementary Figure S1: 2D structures of pre-and pro-vitamin Ds molecules (**A**) Ergocalciferol (pre-vitamin-D_2_) (**B**) Ergosterol (pro-vitamin-D_2_) (**C**) Cholecalciferol (pre-vitamin-D_3_) (**D**) 7-Dehydrocholesterol (pro-vitamin-D_3_) (**E**) 22,23-dihydroergocalciferol (pre-vitamin-D_4_) (**F**) 22,23-Dihydroergosterol (pro-vitamin-D_4_); Supplementary Figure S2: Result of re-docking experiments green colored pose represents original conformation and yellow color represents docked conformation of co-crystalized ligand (A) 6-(ethylamino)pyridine-3-carbonitrile (RZS) of Mpro and (B) 5-amino-2-methyl-N-[(1R)-1-naphthalen-1-ylethyl] benzamide (TTT) for PLpro.
