# Supplementary material for: Deciphering the Potential of Pre and Pro-Vitamin D of Mushrooms against Mpro and PLpro Proteases of COVID-19: An In Silico Approach
Source: Molecules. 2022 Aug 31;27(17):5620. doi: 10.3390/molecules27175620 (PMC9458008; doi:10.3390/molecules27175620)
Supplement: Supplementary file 1 [file molecules-27-05620-s001.zip › molecules-1881276-supplementary.pdf]

<u>**Supplementary Material**</u>

Deciphering the Potential of Pre and Pro-Vitamin D of Mushrooms against Mpro and PLpro Proteases of COVID-19: An In Silico Approach

Abhay Tiwari [1,*], Garima Singh [1], Gourav Choudhir [1], Mohit Motiwale [2], Nidhi Joshi [3], Vasudha Sharma [4,*], Rupesh K. Srivastava [5], Satyawati Sharma [1,*], Marco Tutone [6,*] and Pradeep Kumar Singour [2]

[1] Centre for Rural Development & Technology, Indian Institute of Technology (IIT), New Delhi 110016, India
[2] Computational and Synthetic Chemistry Lab, Department of Pharmaceutical Chemistry, Faculty of Pharmacy, VNS Group of Institutions, Bhopal 462044, India
[3] Department of Pharmacology, University of Minnesota, Minneapolis, MN 55455, USA
[4] Department of Food Technology, Jamia Hamdard, New Delhi 110062, India
[5] Department of Biotechnology, All India Institute of Medical Sciences (AIIMS), New Delhi 110029, India
[6] Dipartimento di Scienze e Tecnologie Biologiche Chimiche e Farmaceutiche, Università degli Studi di Palermo, 90123 Palermo, Italy
* Correspondence: tiwariabhay.19@gmail.com (A.T.); kotpalvasudha@gmail.com or vasudhakatwal@gma (V.S.); satyawatis@hotmail.com or ssharma@rdat.iitd.ac.in (S.S.); marco.tutone@unipa.it (M.T.)

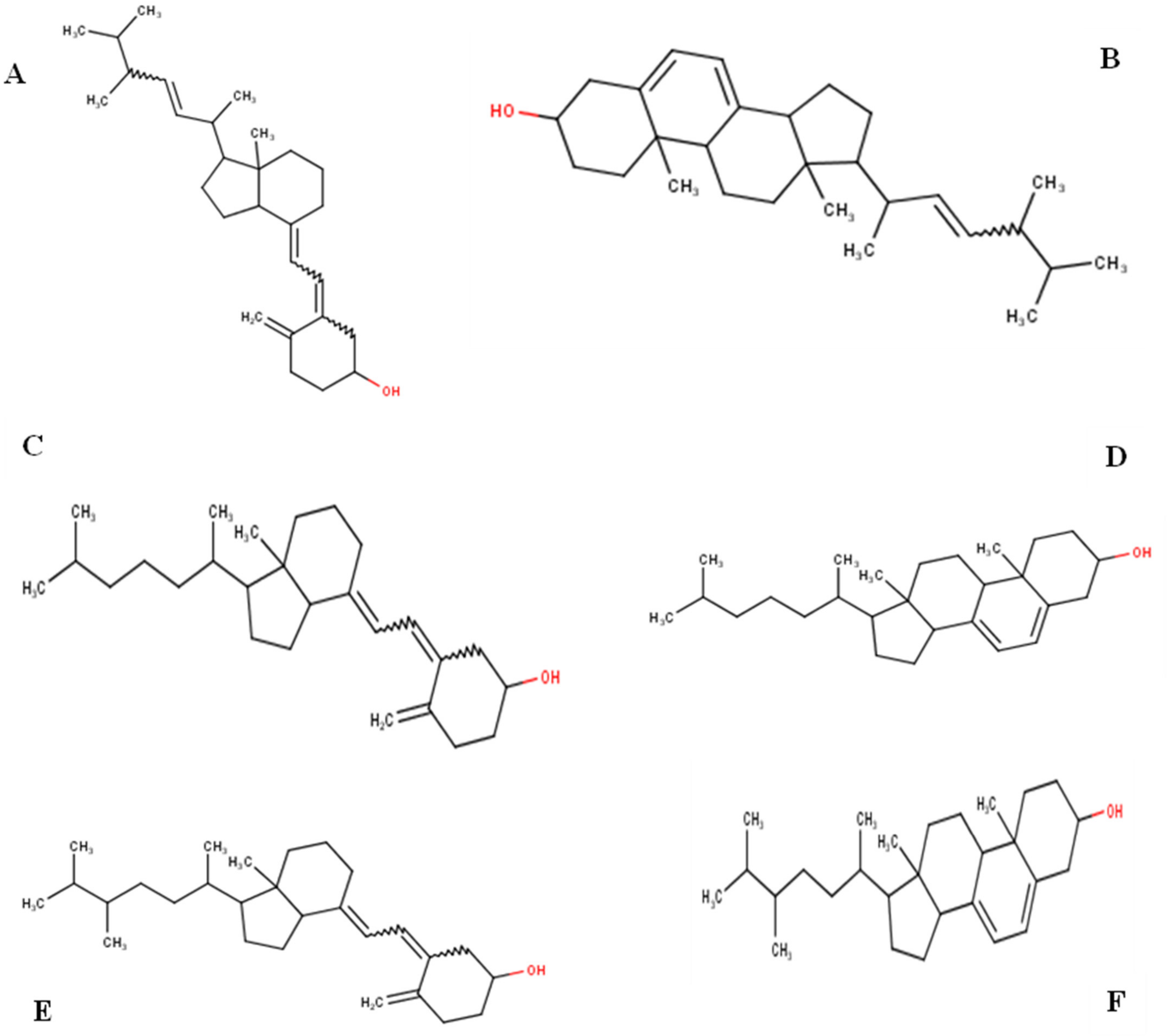

Supplementary Figure S1: 2D structures of pre-and pro-vitamin Ds molecules (**A**) Ergocalciferol (pre-vitamin-D_2) (**B**) Ergosterol (pro-vitamin-D_2) (**C**) Cholecalciferol (pre-vitamin-D_3) (**D**) 7-Dehydrocholesterol (pro-vitamin-D_3) (**E**) 22,23-dihydroergocalciferol (pre-vitamin-D_4) (**F**) 22,23-Dihydroergosterol (pro-vitamin-D_4)

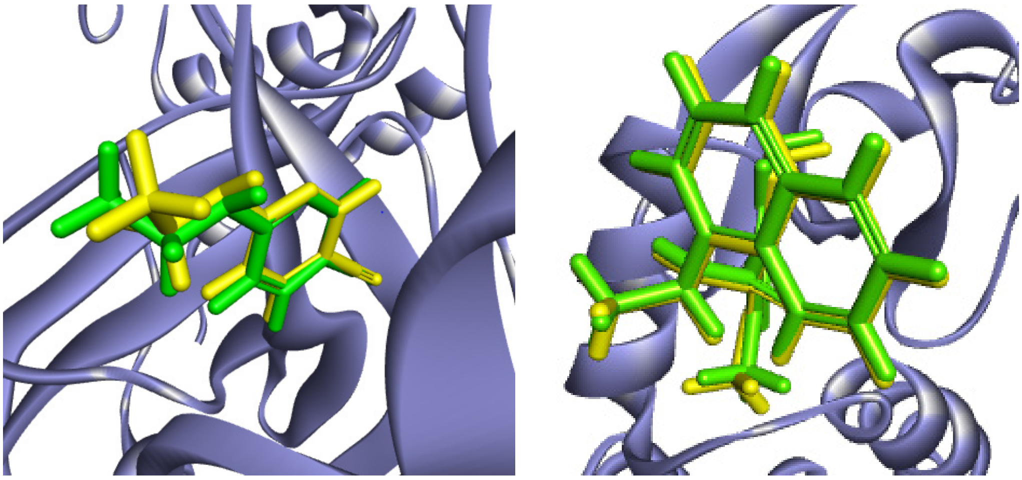

(A) **(B)**

Supplementary Figure S2: Result of re-docking experiments green colored pose represents original conformation and yellow color represents docked conformation of co-crystalized ligand (A) 6-(ethylamino)pyridine-3-carbonitrile (RZS) of Mpro and (B) 5-amino-2-methyl-N-[(1R)-1-naphthalen-1-ylethyl]benzamide (TTT) for PLpro.
